# S-Nitroso-Proteome Revealed in Stomatal Guard Cell Response to Flg22

**DOI:** 10.3390/ijms21051688

**Published:** 2020-03-01

**Authors:** Sheldon R. Lawrence, Meghan Gaitens, Qijie Guan, Craig Dufresne, Sixue Chen

**Affiliations:** 1 Plant Molecular and Cellular Biology Program, University of Florida, Gainesville, FL 32610, USA; s.lawrence@ufl.edu; 2 Department of Biology, University of Florida Genetics Institute, Gainesville, FL 32611, USA; mgaitens@ufl.edu (M.G.); qijie.guan@ufl.edu (Q.G.); 3 Thermo Fisher Scientific, 1400 Northpoint Parkway, West Palm Beach, FL 33407, USA; Craig.dufresne@thermofisher.com; 4 Proteomics and Mass Spectrometry, Interdisciplinary Center for Biotechnology Research, University of Florida, Gainesville, FL 32610, USA

**Keywords:** *Arabidopsis thaliana*, redox proteomics, flg22, iodoTMTRAQ, nitric oxide, stomatal immunity

## Abstract

Nitric oxide (NO) plays an important role in stomata closure induced by environmental stimuli including pathogens. During pathogen challenge, nitric oxide (NO) acts as a second messenger in guard cell signaling networks to activate downstream responses leading to stomata closure. One means by which NO’s action is achieved is through the posttranslational modification of cysteine residue(s) of target proteins. Although the roles of NO have been well studied in plant tissues and seedlings, far less is known about NO signaling and, more specifically, protein S-nitrosylation (SNO) in stomatal guard cells. In this study, using iodoTMTRAQ quantitative proteomics technology, we analyzed changes in protein SNO modification in guard cells of reference plant *Arabidopsis thaliana* in response to flg22, an elicitor-active peptide derived from bacterial flagellin. A total of 41 SNO-modified peptides corresponding to 35 proteins were identified. The proteins cover a wide range of functions, including energy metabolism, transport, stress response, photosynthesis, and cell–cell communication. This study creates the first inventory of previously unknown NO responsive proteins in guard cell immune responses and establishes a foundation for future research toward understanding the molecular mechanisms and regulatory roles of SNO in stomata immunity against bacterial pathogens.

## 1. Introduction

Crop stress due to bacterial pathogens results in sizeable losses in economic revenue annually and threatens global food security [1,2]. Bacteria are unable to penetrate the plant epidermis and enter plants primarily through stomatal pores. They establish themselves in the apoplast where they utilize plants’ nutrient resources and suppress plant immune defense. Stomatal pores are controlled by pairs of specialized guard cells, which swell and shrink as a result of the influx or efflux of ions and water in order to adjust the stomatal aperture in response to environmental stimuli such as light, CO_2_, pathogen and drought [3]. Because of the importance of stomata as a plant’s first line of defense against bacterial pathogens, guard cell signal transduction is essential for bacterial invasion and plant–pathogen interactions.

To rapidly respond to environmental stimuli such as bacterial pathogens, guard cells employ posttranslational modifications (PTMs) of key proteins in signaling pathways [4]. The PTMs of these proteins function as molecular switches to modulate protein functions, thus adding a level of regulation to plant defense processes [5,6]. Increasing studies have been conducted to elucidate the function of PTMs on specific plant proteins [7,8,9,10]. Among the PTMs, S-nitrosylation (SNO) is one of the most common modifications found in plants and its specific involvement in plant stress signaling has been under much investigation over the years [11,12,13].

Nitric oxide (NO) is an essential signaling molecule that functions in myriad physiological processes in plants, including hormone signaling, flowering, growth and development, stomata closure and cell death [14,15,16,17,18]. In plants, NO can be produced via different routes that involve enzymatic or nonenzymatic reactions and a number of different cellular compartments, including chloroplast, mitochondria, peroxisomes, cytosol and plasma membrane [19,20,21,22,23]. The most well understood source of NO is the cytosolic localized nitrite-dependent nitrate reductase pathway. This enzymatic source of NO is produced through the reduction of nitrate to nitrite catalyzed by the enzyme nitrate reductase (NR), which uses NAD(P)H as an electron donor [24]. Nitrite can further be reduced to NO following a reaction similar to that of nitric oxide synthase found in animals. Under normal physiological conditions, NO is present in the cell at low and controlled levels, where it acts as a crucial regulator involved in every stage of normal plant growth and development. However, under abiotic and biotic stresses, NO levels can increase to harmful levels leading to cellular damage. In plants, NO is known to modulate the expression of genes involved in primary metabolism, hormonal signaling and stress responses [25,26].

Over the years, the role of NO as a component of plant immunity against pathogens has become increasingly evident [27,28,29,30]. Pathogen infection triggers NO production. At the early stage of infection, the burst of NO results in S-nitrosothiols that lead to enhanced cell death [31]. However, at later stages the increased levels of NO decrease cell death via nitrosylation of respiratory burst oxidase homolog (RBOH), leading to a reduction in its activity and oxidative stress. This highlights the versatility of NO in the fine-tuning of cell death in response to stress. Additionally, other studies have helped to support NO function in plant defense via SNO. For example, SNO of the transcriptional co-regulator nonexpresser of PR gene 1 (NPR1) at cysteine 156 promotes its localization in the cytoplasm as inactive oligomers [32]. As well, the sucrose non-fermenting-1-related protein kinase (SnRK 2.6)/open stomata 1 (OST1), a positive regulator of abscisic acid (ABA)-induced stomata closure, is regulated via SNO [33]. SNO of OST1 at the cysteine residue (Cys137) abolished the kinase activity, indicating a negative feedback mechanism by which NO helps fine-tune control of ABA signaling in guard cells. In addition, S-nitrosoglutathione reductase 1 (GSNOR1), an important enzyme involved maintaining NO and glutathione levels in the cell, was shown to be regulated by nitrosylation [34]. These studies highlight the importance of SNO in plant defense. However, little is known about NO regulation via protein nitrosylation in guard cell functions. In addition, many redox proteomic studies do not address the issue of protein turnover, which often leads to misleading results of redox regulation [35,36].

In an effort to gain a greater understanding of the role of nitrosylation in plant responses to bacterial stress and guard cell signaling, we have employed a quantitative proteomics approach in analyzing the S-nitroso-proteome of *Arabidopsis thaliana* guard cells. Using a double-labeling strategy (iTRAQ and iodoTMT) termed iodoTMTRAQ, in which redox changes such as SNO and protein level changes can be monitored in one experiment, we identified a total of 41 SNO-modified peptides, which corresponded to 35 SNO-responsive proteins, and they were significantly changed in response to flg22 treatment. The proteins function in energy metabolism, transport, stress response, photosynthesis, and cell–cell communication. These results reveal an inventory of SNO-responsive proteins and their associated pathways in guard cell flg22 responses and will contribute to our understanding of molecular mechanisms underlying guard cell pathogen signaling.

## 2. Results and Discussion

### 2.1. Enriched Arabidopsis Guard Cells Are Viable

**Figure 1:**
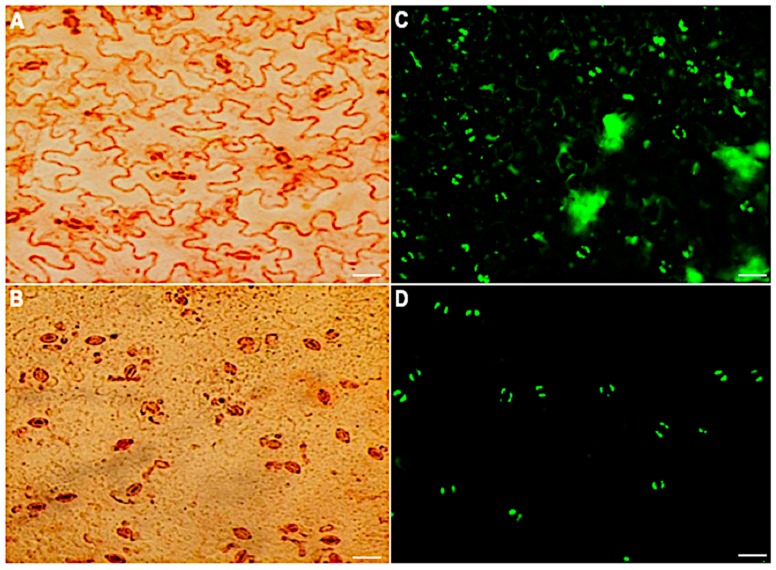
Stomatal guard cell purity and viability before and after the removal of mesophyll and epidermal cells. Neutral red viability staining of epidermal peels: (**A**) before digestion and (**B**) after digestion. FDA viability staining of the peels: (**C**) before digestion and (**D**) 60 min after digestion. Scale bar: 30 µm.

To maintain the stomatal movement output that enables morphological, physiological and other biological studies, a method for isolating intact stomatal guard cells rather than guard cell protoplasts was developed. In this method, *A. thaliana* leaves were partitioned between two portions of transparent Scotch tape and peeled apart to separate the abaxial side. The abaxial side of the peels was transferred to an enzyme solution to digest away the epidermal cells. After 20 minutes of digestion, the stomatal guard cells were left to recover for 60 min under light. Peels were then assessed for guard cell viability and purity using neutral red and fluorescein diacetate (FDA), respectively. The guard cells were the dominant cell type ( > 90%) on the peels and were intact and viable (Figure 1).

### 2.2. Flg22 Induction of Stomatal Closure and Production of Reactie Oxygen Species (ROS) and NO

**Figure 2:**
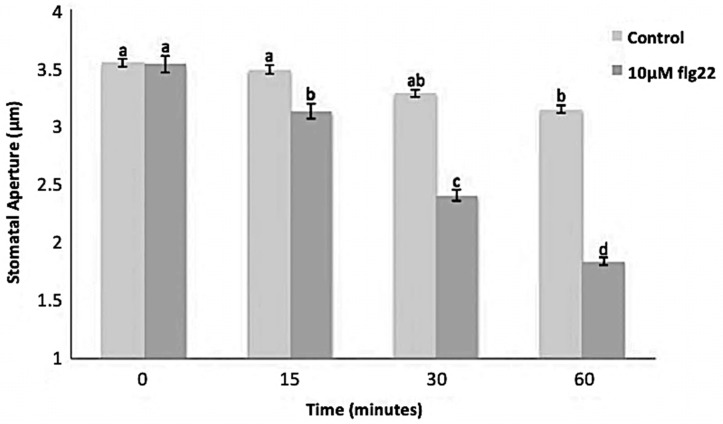
Stomatal movement in response to 10 μM flg22. Data were obtained from 180 stomata from three independent experiments and presented as means ± SE. Different letters indicate significantly different mean values at *p* < 0.05.

**Figure 3:**
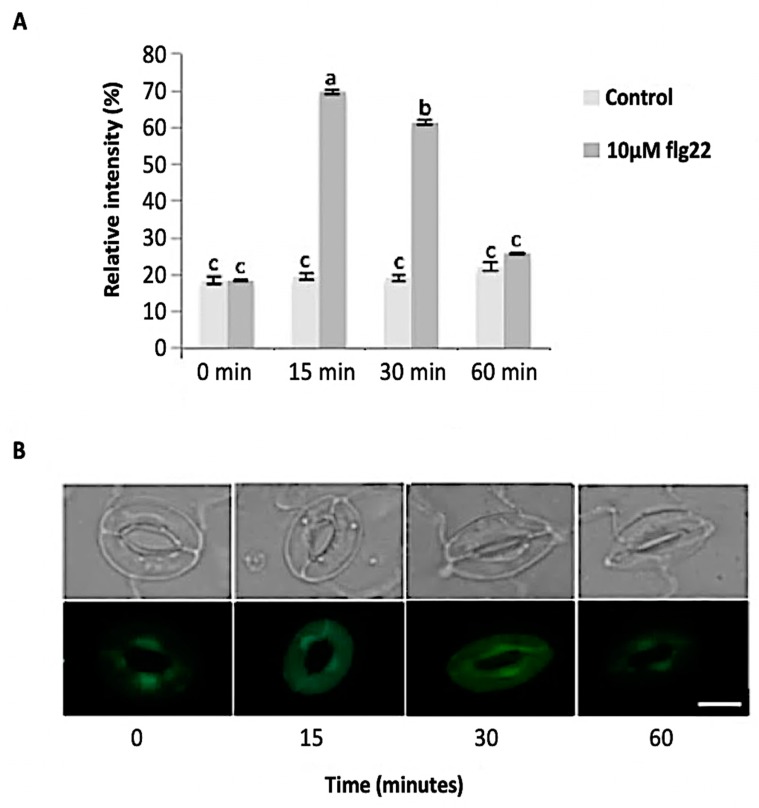
Reactive oxygen species (ROS) sproduction in guard cells in response to 10 μM flg22. (**A**) ROS levels measured from a total of 180 stomata from three independent experiments and presented as means ± SE. Different letters indicate significantly different mean values at *p* < 0.05. (**B**) Representative images of stomatal guard cells at each time point are shown. Scale bar: 10 µm.

**Figure 4:**
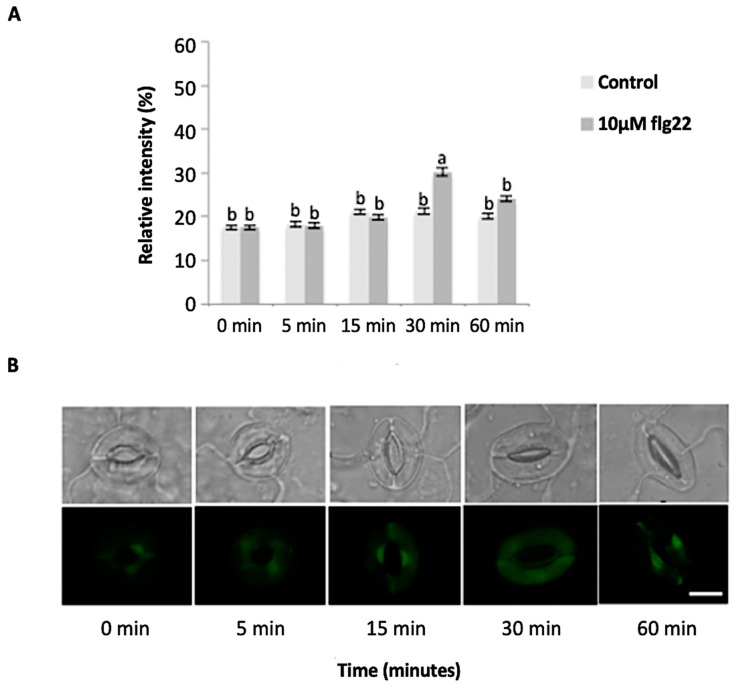
Guard cell nitric oxide (NO) levels in response to 10 µM flg22. (**A**) NO levels measured from a total of 180 stomata from three independent experiments and presented as means ± SE. Different letters indicate significantly different mean values at *p* < 0.05. (**B**) Representative images of stomatal guard cells at each time point are shown. Scale bar: 10 µm.

The bacterial flagellin peptide flg22 is a well-known plant defense elicitor that induces stomatal closure [37]. To test whether this response was present in the enriched *A. thaliana* guard cells using the above method (Figure 1), 10 μM flg22 was added to the guard cell peels and the stomatal aperture was monitored. Upon flg22 treatment, stomata begin closing as early as 15 min after treatment, and after 60 min stomata aperture was decreased by more than half (Figure 2). Next, the ability of the enriched stomatal guard cells to produce ROS and NO was tested. The redox indicator compounds H_2_DCF-DA and DAF-2DA were used to measure ROS and NO levels, respectively. We show that 10 μM flg22 treatment induced ROS (Figure 3) and NO production (Figure 4) in the enriched stomatal guard cells. Guard cells were able to produce ROS and NO with the highest levels at 15 minutes and 30 minutes, respectively, after the treatments. These results suggest that ROS, NO, and/or overall guard cell redox state play significant roles in guard cell signaling that leads to stomatal closure. Furthermore, they show that guard cell molecular and biochemical responses remain intact in the enriched guard cell peels.

### 2.3. Identification of flg22-Regulated Proteins and Nitrosylated Proteins

To identify nitrosylated proteins and uncover their roles in flg22-triggered stomatal closure, we used a double-labeling strategy with iodoTMT and iTRAQ, which allows for simultaneous analysis of cysteine redox changes and total protein level change [38,39]. In this strategy, we conducted redox proteomic analysis of control and treatment samples at 15, 30, and 60 min time points. Proteins were extracted in the presence of N-ethylmaleimide (NEM) to irreversibly block free thiols and prevent artificial cysteine oxidation during protein extraction. Then, the nitrosylated cysteine residues were reduced with sodium ascorbate, and the protein thiols were labeled with iodoTMT tags for quantification. The iodoTMT proteins were digested with trypsin and the peptides were labeled with iTRAQ tags. This reverse-labeling strategy allows for the maintenance of the redox state of the proteins after treatment and prevents artificial oxidation during sample preparation. Therefore, the differences in iodoTMT signals from specific peptides derived from treated samples compared to control samples indicate the presence of redox-sensitive nitrosylated cysteine residues. Furthermore, the differences in iTRAQ tags from control compared to treatment samples indicate the change in total protein level between the two during the stomatal closure process. In this study, a total of 433, 578, and 463 unique cysteine-containing peptides corresponding to 229, 283, and 237 proteins were confidently identified (FDR 0.01) among three biological replicates of the samples treated with 10 μM flg22 for 15, 30, and 60 min, respectively. In order to determine the flg22-redox sensitive cysteines, we first compared the relative peak intensity in the control and treatment samples. A *p*-value smaller than 0.05 and a threshold of greater than 1.2 or less than 0.8 were set as criteria to determine significant differences between the control and treatment. A total of 41 SNO modified peptides corresponding to 6, 12, and 21 potential NO regulated redox proteins were identified at 15, 30, and 60 min, respectively. Among these proteins, 6, 2, and 17 underwent oxidation, while 0, 10, and 4 underwent reduction after flg22 treatment at 15, 30, and 60 min, respectively (Table 1).

As previously stated, redox-regulated proteins can be challenging to identify due to protein level changes. With the double-labeling strategy, in addition to monitoring redox changes we were able to monitor changes in protein levels. In total, 2614, 3804, and 3327 peptides corresponding to 1429, 2012, and 1745 proteins were confidently identified. When the control and treatment samples were compared via iTRAQ labels, which quantify protein level changes using the same criteria as iodoTMT, a total of 9, 28, and 41 proteins were identified to be significantly changed in samples treated with flg22 at 15, 30, and 60 min, respectively (Supplemental Table S1–S3). Of these 7, 16, and 31 were shown to increase while 2, 12, and 10 decreased at 15, 30, and 60 min after treatment respectively. In this study, iTRAQ data was used to determine if the potential redox-responsive proteins identified by iodoTMT were in fact due to redox changes between the control and treated samples or a result of protein level changes. After considering the protein level changes, 6, 10, and 19 redox proteins were determined to be bona fide nitrosylated proteins that were responsive to the flg22 treatment at 15, 30, and 60 min, respectively.

#### 2.3.1. Guard Cell S-nitroso-proteomic Changes in Response to flg22 at Early Stage of Stomatal Closure

In this study, early stomatal responses were defined at the 15- and 30-minute time-points. They reflect the highest levels of ROS and NO, respectively. At 15 minutes after flg22 treatment, there were seven nitrosylated proteins identified. They include curculin-like lectin family protein, mannose binding lectin protein, bifunctional inhibitor/lipid-transfer protein/seed storage 2S albumin, vacuolar H^+^-ATP synthase subunit E1, nitrilase 1 (NIT1), S-adenosyl-l-homocysteine hydrolase 2 (SAHH2), and chlorophyll B-binding protein (Table 1). Analysis of gene ontology (GO) terms revealed that these proteins are involved in protein transport, carbohydrate binding, and nitrogen compound metabolic process. It should be noted that several other proteins were initially thought to be redox-regulated. However, after protein fold change was taken into account, those proteins were found to show significant protein level change. Therefore, the observed redox-fold change in those may have been a result of change in protein levels rather than the cysteine redox response, thus they were discarded as potential nitrosylated proteins. There were nine cysteine-containing peptides from the seven different proteins that showed changes in redox state among control and treated samples. Four of the nine peptides derived from two proteins (D-mannose binding lectin protein and bifunctional inhibitor/lipid-transfer protein/seed storage 2S albumin), while the other five derived from only a single peptide. The subcellular localization of the six proteins was predicted using both internet (YLoc and LocTree3) and literature information. In total, five of the six proteins were predicted to be localized to the cell wall or plasma membrane in addition to one or two different organelles. Individual analysis revealed that five of these proteins, D-mannose binding lectin protein, SAHH2, vacuolar H^+^-ATP synthase subunit E1, NIT1, and curculin-like lectin family protein were previously reported to undergo S-nitrosylation following salt or cold stress [40,41,42]. The sequences of the identified nitrosylated proteins were analyzed for possible intra-molecular disulfide bond formation using DiANNA software (http://clavius.bc.edu/~clotelab/DiANNA/). All six of the redox-sensitive proteins were predicted to form intra-molecular disulfide bonds (Table 1). A few of the predicted cysteine(s) were the same as those identified as having the SNO modification. It is important to note that disulfide bond formation is another possible modification. However, in this study we detected SNO modification instead of disulfides.

**Figure 5:**
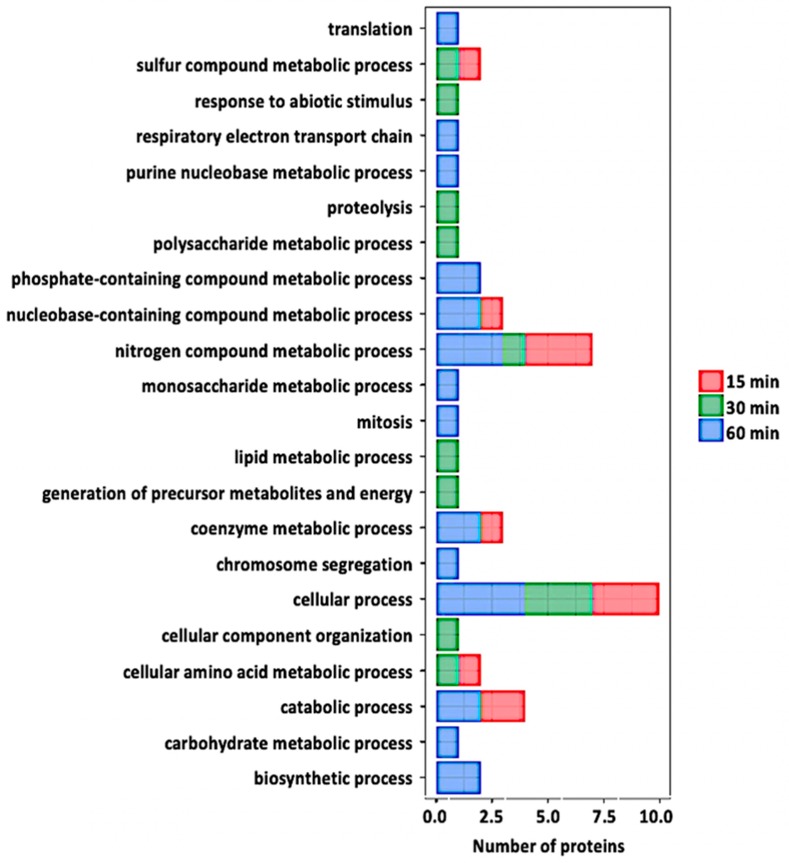
Gene ontology analysis of the redox proteins after flg22 treatment. Relevant biological processes are shown on the *y*-axis. Number of proteins significantly redox-regulated at each time point after treatment is shown on the *x*-axis.

At 30 min post treatment, 12 nitrosylated proteins were identified. Interestingly, only two of them (glutathione S-transferase phi 12 and bifunctional inhibitor/lipid-transfer protein/seed storage 2S albumin) were found to be in oxidized states, while the other ten proteins (fK506-binding protein, beta-hexosaminidase, d-mannose binding lectin protein, RmlC-like cupins, S-adenosyl-L-methionine-dependent methyltransferase, glycosyl hydrolase family 35 protein, eukaryotic aspartyl protease, cruciferin, chlorophyll B-binding protein, and Beta-carbonic anhydrase) were in a reduced state compared to the control samples (Table 1). Two proteins (d-mannose binding lectin protein and bifunctional inhibitor/lipid-transfer protein/seed storage 2S albumin) were redox-regulated at both 15- and 30-min time points. Analysis of protein level changes revealed that neither of the proteins showed significant protein level changes. Analysis of potential intra-molecular disulfide bond formation revealed that eleven of the twelve potentially nitrosylated proteins were predicted to form intra-molecular disulfide bonds (Table 1). Here, several of the predicted cysteine(s) were the same as those identified as having the SNO modification. Biological processes of these potential redox-regulated proteins include lipid metabolic process, photosynthesis, and nitrogen compound metabolic process (Figure 5). Further analysis revealed that the SNO-proteins identified were localized to the chloroplast, cytoplasm, cell wall, or vacuole with the majority showing localization to the chloroplast and having diverse functions in photosynthetic electron transfer and carbon utilization. In addition, several, including bifunctional inhibitor/lipid-transfer protein/seed storage 2S albumi, glutathione S-transferase, mannose-binding lectin protein, chlorophyll B-binding protein, and Beta-carbonic anhydrase, have been implicated in stomatal movement in response to stress [43,44,45,46,47]. This suggests that these proteins have important roles in stomatal function that may be regulated via S-nitrosylation. In addition, these findings are in agreement with the idea that photosynthesis is highly redox-regulated [48], and it is interesting to know that the regulation in guard cells is via protein nitrosylation.

#### 2.3.2. Guard Cell S-nitroso-proteomic Changes in Response to flg22 at Late Stage of Stomatal Closure

At 60 min of flg22 treatment, 21 nitrosylated proteins were identified (Table 1). Seventeen proteins (oxidoreductase/zinc-binding dehydrogenase, cell division cycle 48, adenosine kinase 1, GPI-anchored adhesin-like protein, ATP synthase, NAD (P)-binding protein, ribulose-bisphosphate carboxylase (Rubisco) small subunit, Rubisco large subunit, acetylcoenzyme A carboxylase 1, cysteine proteinase, glycine-rich protein 2B, aspartate aminotransferase 1, PSI iron-sulfur center subunit PsaC, glycine-rich protein 3, vacuolar H^+^-ATP synthase subunit E1, NIT1, and SAHH2) were in oxidized states, whereas the other four (carbonic anhydrase 1 and 2, 2-phosphoglycolate phosphatase 1, and glyceraldehyde-3-phosphate dehydrogenase (GAPDH)) were in reduced states compared to control samples (Table 1). It is worth mentioning that carbonic anhydrase was identified to be reduced in both 30 and 60 minutes after the treatment. In addition, NIT1 and SAHH2, previously reported to undergo protein nitrosylation in *Arabidopsis* seedlings [31], were identified in guard cells and were oxidized at both early and late stages of stomatal closure. None of these proteins showed significant protein level changes. Analysis of potential intra-molecular disulfide bonds revealed that 17 of the 21 potential nitrosylated proteins were predicted to form intra-molecular disulfide bonds. Again, several of the predicted cysteine(s) were the same as those identified as having the SNO modification. Analysis of GO terms revealed that the biological processes of the 21 potentially nitrosylated proteins include defense response, transcription, carbohydrate metabolic process, response to ABA, proteolysis, oxidation–reduction process, response to hypoxia, lipid metabolic process, and electron transport, amino acid biosynthetic process (Figure 5).

#### 2.3.3. Proteomic Changes in the Course of flg22-induced Stomatal Closure

Stomatal closure was significant at as early as 15 minutes after flg22 treatment. At 15 min, seven proteins showed a significant increase in expression levels after treatment (Supplemental Table S1). These proteins are involved in cellular processes of cell wall biogenesis/degradation, oxidoreductase process, lipid catabolic process, and stomata regulation. Only two of the total significantly changing proteins showed decreased levels after the treatment. They were light harvesting complex photosystem II (PSII) subunit 6 and chlorophyll a-b binding protein 3, which function in the energy capture process of photosynthesis. The small number of proteins that showed significant changes at 15 minutes after treatment correlates with the number identified to be potentially redox-regulated. At 30 min, 16 proteins showed increased expression levels (Supplemental Table S2). The proteins fell into biological processes that include oxidative–reductive process (malate dehydrogenase 1, bifunctional dTDP-4-dehydrorhamnose 3,5-epimerase/dTDP-4-dehydrorhamnose reductase, pyruvate dehydrogenase E1 subunit alpha-3, formate dehydrogenase, GAPDH, isocitrate dehydrogenase, UDP-glucose 6-dehydrogenase 3, 3-ketoacyl-CoA thiolase 2, and citrate synthase 4), response to cytokinin (60S acidic ribosomal protein P0-2 and ABC transporter B4), response to salt stress (malate dehydrogenase 1, 40S ribosomal protein Sa-1, 60S acidic ribosomal P0-2, isocitrate dehydrogenase, and ras-related protein). It is clear that most of the proteins with increased levels belong to the oxidative–-reductive process. These proteins may be critical to maintaining guard cell redox homeostasis in response to stresses. The 12 proteins showing decreased levels after flg22 treatment are involved in processes that include photosynthesis (light harvesting complex PSII subunit 6, PSII CP47 reaction center protein, and cytochrome b6), metabolic energy generation (fructose-bisphosphate aldolase 5, isocitrate dehydrogenase catalytic subunit 6, PSII CP47 reaction center protein, and cytochrome b6), oxidation–reduction process (isocitrate dehydrogenase subunit 6, glutamate dehydrogenase-1, peroxidase, and thioredoxin M4) (Supplementary Table S2).

At 60 min after flg22 treatment, 31 proteins with increased levels were identified (Supplementary Table S3). Analysis of the biological processes revealed that many of the proteins are involved in plant response to stress. For example, leucine-rich repeat (LRR) protein, dehydrin ERD10, 3-ketoacyl-CoA thiolase 2, calcium-dependent protein kinase 9, ascorbate peroxidase 1, and abscisic acid receptor PYL2 are responsive to biotic stress and endogenous stimuli. In addition, many proteins belong to the oxidation-reduction process, and they include GAPDH, malate dehydrogenase 1, succinate dehydrogenase [ubiquinone] iron-sulfur subunit 1, glutamate dehydrogenase 2, and cytochrome P450. Other proteins that showed increases after flg22 treatment are involved in biological processes such as carbohydrate metabolic process (biotin carboxyl carrier protein of acetyl-CoA carboxylase 1, beta-D-xylosidase 1, cellulose synthase A catalytic subunit 1, malate dehydrogenase 1, and GAPDH), as well as cellular process (clathrin light chain 1, calmodulin-like protein 12, cytochrome b6-f complex iron-sulfur subunit). Only a small portion of the identified proteins showed decreased levels at 60 min after flg22 treatment, and they are categorized mostly into metabolic process (beta-D-glucopyranosyl abscisate beta-glucosidase, calreticulin-3, bifunctional 3-dehydroquinate dehydratase/shikimate dehydrogenase, chlorophyll a-b binding protein, transportin-1, fructose-bisphosphate aldolase 1, and cytosolic enolase 3) (Supplementary Table S3).

### 2.4. Functional Classification of Nitrosylated Proteins

To explore the regulatory roles of protein nitrosylation in specific biological processes, we performed GO analysis to functionally classify the S-nitrosylated proteins identified in this study (Figure 5). We identified 35 nitrosylated proteins that covered a wide range of biological processes, including metabolism, transport, stress response, photosynthetic and other cellular processes. Among the 35 proteins, the most enriched category was related to several metabolic processes, suggesting that metabolism is actively regulated by SNO. As previously stated, the role(s) of a number of the proteins identified in this study have been described in stomatal guard cell function during stress, thus it would be interesting to know whether SNO is central to their role(s).

**Figure 6:**
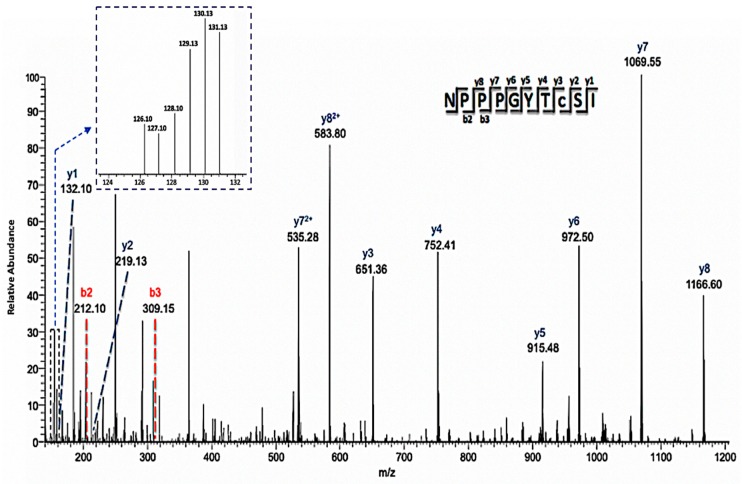
Annotated mass spectrum of a representative cysteine-containing peptide (C8-iodoTMT) showing differential redox status from control and treated samples. The MS/MS ions used to identify the peptide were labeled, and the intensity of the individual iodoTMT peaks for quantification was inserted in the upper left corner. This peptide is one of two that was used to identify lipid-transfer protein isoform II as a potential NO regulated protein.

**Figure 7:**
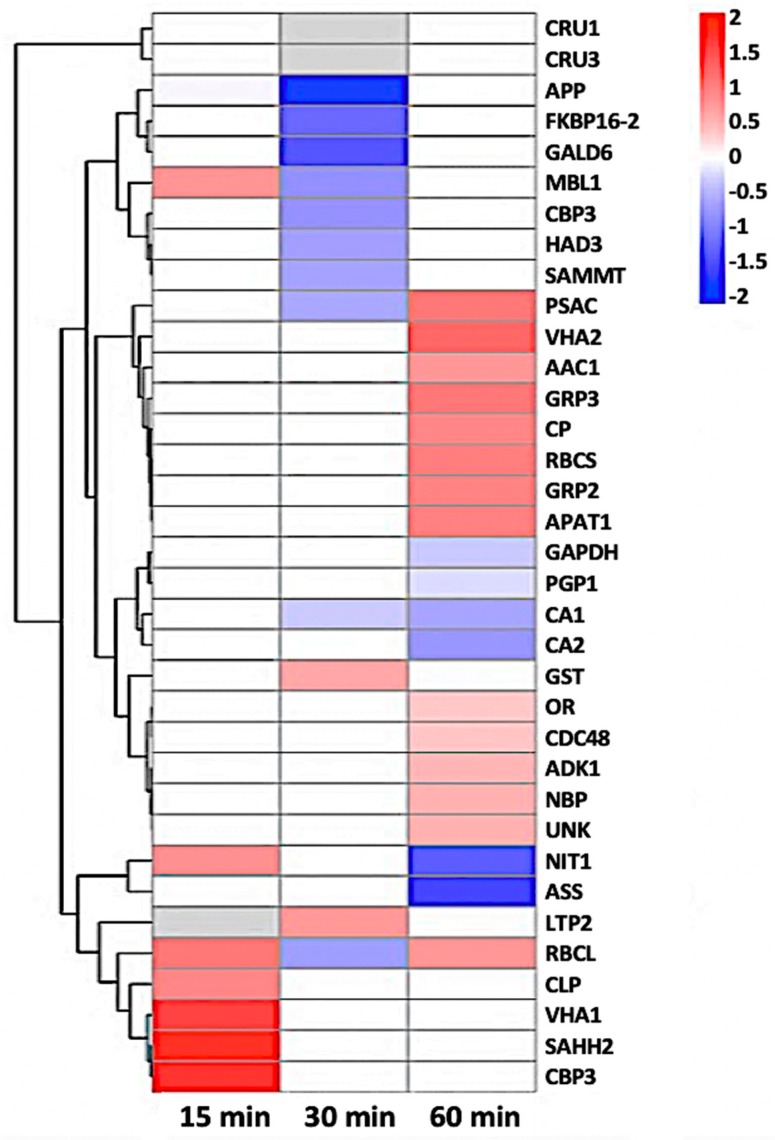
Heat-map of 35 nitrosylated proteins obtained by hierarchical clustering. The columns represent different time point ratios of treatment/control. The rows represent individual proteins. Protein AGI numbers are listed to the right. The increased and decreased proteins are represented in red or green, respectively. The color intensity increases with increasing differences, as shown in the scale bar. Please refer to Table 1 for detailed information.

In addition, a significant number of proteins involved in plant response to stresses were identified to be nitrosylated. These proteins include oxidoreductase, beta-carbonic anhydrase, cruciferin 1 and 3, glycine-rich protein 2, adenosine kinase 1, glutathione S-transferase phi 12, and a curculin-like lectin. Interestingly, a lipid transfer protein LTP-II was identified by two unique peptides as a potential NO-regulated protein (Figure 6). However, its role in plant defense is unclear and future studies to elucidate its role are necessary. We also identified SAHH2 and NIT1, two NO-regulated proteins known to be involved in nitric oxide metabolism and regulating the cellular redox state [41]. This suggests the involvement of redox signaling in the stomatal immune responses. To gain insight to the dynamics of nitrosylation in the time-course experiments, a heat-map of the 35 nitrosylated proteins was generated (Figure 7). As previously stated, the majority of the proteins belonged to metabolic processes. Here a large number were involved in primary metabolism such as carbohydrate, amino acid, and lipid processes. Although no distinct pattern was observed between the time points, it was clear that SNO is represented in many guard cell processes and reveals increases in protein regulation as early as 15 minutes after flg22 treatment. This further provides support that SNO is an important PTM in stomatal responses to bacterial pathogens. In addition, several proteins revealed by this study were previously functionally characterized as NO targets, suggesting the utility and reliability of this iodoTMTRAQ method in the identification of NO target proteins. Despite the identification of many previously unknown NO responsive proteins in guard cells, additional studies will be needed to build a functional network of NO signaling in stomatal immunity against bacterial pathogens. Based on the results presented, it can be reasoned that many of the identified proteins may be important players in guard cell perception of NO during pathogen infection.

## 3. Materials and Methods

### 3.1. Plant Material

*Arabidopsis thaliana* ecotype Columbia (Col-0) seeds were obtained from the Arabidopsis Biological Resource Center (ABRC, Columbus, OH, USA). Seeds were germinated in a Metro-Mix 500 potting mixture (The Scotts Co., Marysville, OH, USA). The seedlings were grown in a growth chamber at 140 µmol photons m^−2^s ^−1^ with a photoperiod of 8-hour light at 22 °C and 16-hour dark at 18 °C for 5 weeks. Fully expanded leaves were used for stomatal movement assay and guard cell enrichment.

### 3.2. Preparation of Epidermal Peels for Stomatal Movement Assay

Epidermal peels were prepared as previously described with minor modifications [49]. Small squares (0.5 × 0.5 cm^2^) of leaf sections from the 5-week-old *A. thaliana* plants were fixed abaxial side down onto coverslips coated with a medical adhesive (Hollister, Libertyville, IL, USA). Adaxial epidermis and mesophyll layers were removed with a scalpel. After washing twice with distilled water to remove cellular debris, the coverslips were incubated with a cell wall digesting enzyme mixture at 26 °C, 140 excursions per min on a reciprocal shaker for 15 min. The enzyme mixture was constituted as follows: 0.7% cellulase R-10 (Yakult Honsha Co., Ltd, Tokyo, Japan), 0.025% macerozyme R-10 (Yakult Honsha Co., Ltd, Tokyo, Japan), 0.1% (*w/v*) polyvinylpyrrolidone-40 (Calbiochem, Billerica, MA, USA), and 0.25% (*w/v*) bovine serum albumin (Research Products International Corp., Mt Prospect, Illinois, USA) in a basic solution (0.55 M sorbitol, 0.5 mM CaCl_2_, 0.5 mM MgCl_2_, 0.5 mM ascorbic acid, 10 μM KH_2_PO_4_, 5 mM 4-morpholineethanesulfonic acid (MES), pH 5.5 adjusted with 1 M KOH). The coverslips were then incubated in an opening buffer (10 mM KCl, 50 μM CaCl_2_, 10 mM MES-KOH, adjusted to pH 6.15 with 1 M KOH) under light (110 μmol m^−2^s^−1^) for an hour. For flg22 experiments, the stomata were treated with either 10 μM flg22 or water (mock). A total of 60 stomatal apertures for each individual experiment were measured with imaging software Image J version 1.50 (NIH, USA). Three independent experiments were conducted. Standard error and significance at a *p*-value < 0.05 were calculated using Microsoft Excel.

### 3.3. Stomatal ROS and NO Measurement

For ROS measurement, 50 μM of the redox-sensitive 2′-7′-dihydro-dichlorofluorescein diacetate (H_2_DCF-DA) was added to the opening buffer and incubated the epidermal peels for 30 min. After washing with opening buffer to remove excess dye, the epidermal peels were transferred to a Petri dish containing water (control) or water with 10 μM flg22 (treatment). Images of stomata were captured using the Leica DM 6000 B fluorescence microscope (Leica, Buffalo Grove, IL, USA) with excitation 450–490 nm and emission 500–550 nm. For NO measurement, the same procedure was followed using a NO-sensitive fluorescent dye 4,5-diamino fluorescein diacetate (DAF-2DA) with excitation 495 nm and emission 515–560 nm. The fluorescence emission levels of 150 stomata from three independent experiments were analyzed using ImageJ software (National Institutes of Health, Bethesda, MD, USA).

### 3.4. Large-Scale Preparation of Stomatal Guard Cells for Proteomics

Stomatal guard cells from *A. thaliana* plants were prepared using a Scotch tape method as previously described [50]. Briefly, 5-week-old leaves were attached to clear Scotch tapes (3M, St Paul, MN, USA) with the abaxial side facing down. Another piece of tape was used to separate the adaxial layer containing the mesophyll cells from the abaxial side containing pavement and guard cells. A total of 100 epidermal peels constituted a biological replicate, and three replicates were collected. The peels were digested to remove the pavement cells as aforementioned. The samples were treated with 10 μM flg22 or with water and collected at 0, 15, 30, and 60 min. The samples were quickly frozen in liquid nitrogen and stored at −80 °C before protein extraction.

### 3.5. Protein Extraction

The enriched guard cells were ground in liquid nitrogen. For every 100 peels, 6 mL of Tris saturated phenol (pH 8.8) and 6 mL protein extraction buffer (0.9 M sucrose, 0.1 M Tris-HCl, 0.01 M EDTA, 1 mM PMSF, and 20 mM NEM were added and the peels were ground for 5 mins in a fume hood. The homogenate was transferred to Oakridge centrifuge tubes (Thermo Fisher Scientific, Waltham, MA, USA) and agitated on a shaker at room temperature (RT) for 1 h, followed by centrifugation at 15,000 g at 4 °C for 15 min. The supernatants were added to 5 volume of 100 mM ammonium acetate/methanol and kept at −20 °C overnight. After centrifugation at 15,000 g for 15 min at 4 °C, the pellets were washed with 100 mM ammonium acetate/methanol twice, cold 80% acetone twice, and 100% acetone once. The pellets were dissolved in a protein dissolution buffer (6 M urea, 1 mM EDTA, 50 mM Tris-HCL (pH 8.5) and 1% SDS). Protein samples were prepared from three independent biological replicates, each containing 100 peels.

### 3.6. iodoTMT Labeling and Digestion

The iodoTMT labeling of thiols was performed as described previously [51]. Reduced thiols for reverse labeling were generated by incubation with 10 mM sodium ascorbate (to specifically reduce SNO-modified cysteine residues) at RT for 1 h. For this study we labeled the 15-, 30-, and 60-min control samples with 126, 127, and 128 mass tags, and the flg22-treated samples with 129, 130, and 131 mass tags, respectively. Labeling was performed with protection from light for 2 h at 37 °C. The reaction was quenched with 0.5 M DTT for 15 min at 37 °C. The samples were then separated on a 12% SDS-PAGE gel at 100 V for 5 min and then 80 V for 10 min. This step is to clean up the protein samples and prepare them for in-gel digestion with trypsin. Trypsin digestion was performed at 37 °C overnight as recommended by the iodoTMT manual (Thermo Scientific, San Jose, CA, USA). Excess trypsin was removed, and peptides were cleaned up using C18 desalting columns (Thermo Scientific, San Jose, CA, USA) and lyophilized to dryness.

### 3.7. iTRAQ Labeling, Strong Cation Exchange Fractionation and Reverse Phase LC-MS/MS

For iTRAQ labeling, the lyophilized samples were resuspended in 0.5 M triethylammonium bicarbonate. The peptides were labeled with the iTRAQ reagents according to the manufacturer’s manual (AB Sciex Inc., Framingham, MA, USA). A final concentration of 70% isopropanol was used for all the iTRAQ tags. We labeled the 15-, 30-, and 60-min control samples with 113, 114, and 115 mass tags, and the flg22-treated samples with 116, 117, and 118 mass tags, respectively. The labeling was carried out at 37 °C overnight. Labeled peptides were desalted with C18-solid phase extraction and dissolved in strong cation exchange (SCX) solvent A (25% *v/v* acetonitrile, 10 mM ammonium formate, and 0.1% *v/v* formic acid, pH 2.8). The peptides were fractionated using an Agilent high performance liquid chromatography (HPLC) 1260 with a SCX column (polysulfoethyl A 2.1 mm, 100 mm, 5 μm, 300 Å). Peptides were eluted with a linear gradient of 0% to 20% solvent B (25% *v/v* acetonitrile and 500 mM ammonium formate, pH 6.8) over 50 min followed by ramping up to 100% solvent B in 5 min. Peptide absorbance at 280 nm was monitored, and 20 fractions were collected, followed by desalting and lyophilization.

The fractionated peptides were resuspended in loading solvent A (3% *v/v* acetonitrile, 0.1% *v/v* acetic acid) and separated on an EASY-nLC1000 system coupled to a Q-Exactive Orbitrap Plus^TM^ mass spectrometer (Thermo Scientific, Bremen, Germany). The peptides were loaded onto a C18 PepMap nanoflow column (20 mm × 75 μm; 3 μm-C18), and then separated on a PepMap RSLC EASY-column (250 mm × 75 μm; 2 μm-C18). The elution gradient started at 97% solvent A (0.1% *v/v* acetic acid, 3% *v/v* acetonitrile)/3% solvent B (0.1% *v/v* acetic acid, 96.9% *v/v* acetonitrile) and finished at 40% solvent A/60% solvent B within 90 min. After each run, the column was washed with 90% solvent B and re-equilibrated with solvent A. MS/MS analysis was carried out in positive mode, applying data-dependent MS scanning and MS/MS acquisition (Thermo Scientific, Bremen, Germany). The chromatographic peak width was 4 s and the default charge state was 3. Survey full-scan MS spectra scan range was 400–2000 m/z, with a resolution of 70,000 at 200 m/z. The MS/MS resolution was 17,500. The first mass was fixed at 105 m/z to accommodate the lower m/z iTRAQ reporter ions and 445.12003 m/z (polysiloxane ion mass) was used for real-time mass calibration.

### 3.8. Database Searching and Data Analysis

Proteome Discoverer (PD) 2.2 (Thermo Scientific, Bremen, Germany) was used for protein identification based on searching the raw data against the uniprot *A. thaliana* database. PD nodes for spectrum grouper and spectrum selector were set to default with the spectrum properties filter set to a minimum and maximum precursor mass of 300 DA and 5 kDa, respectively. The SEQUEST HT algorithm was used for protein identification. Parameters were set to two maxima missed cleavage sites of trypsin digestion, absolute XCorr threshold of 0.4, and fragment ion cutoff percentage at 0.1. Tolerances were set to a 10 ppm precursor mass tolerance and a 0.02 Da fragment mass tolerance. Dynamic modifications included phosphorylation (+79,966 (S, T, Y)), oxidation (+15.995 Da (M)), N-ethylmaleimide (+124.048 Da (C)), iTRAQ8plex (+304.205 Da (N-terminus and K)), and iodoTMT6plex (+329.227 Da (C)). Percolator was used for protein identification with parameters of a strict target false discovery rate (FDR) of 0.01 and a relaxed target FDR of 0.05. The reporter ion quantifier node was set with a peak integration tolerance of 20 ppm, and the event detector used mass precision set to 2 ppm with a signal-to-noise ratio threshold of 3. Quantification was preformed using the iodoTMT and iTRAQ reporter ion peak intensities. Peptides were filtered to include only those with high confidence of identification (1% FDR).

Unique peptides were used for the relative protein quantification. Peak intensities for each iodoTMT label (126–131) were exported, and ratios were calculated accordingly from the median-normalized peak intensity values. Student’s t-test (two-tailed) on the log2-transformed treated/control ratios was performed. To control false discovery rates, Benjamini–Hochberg correction of *p*-values [52] was performed by using p.adjust function in the R-package. A peptide with a *p*-value 0.05 was considered to be statistically significant. The iTRAQ data were searched using the same database to generate information on protein level changes. For the iTRAQ data, peptides were normalized to the total summation of their intensities based on the 113-reporter channel. Protein grouping was performed by taking the sum of repeated peptides followed by median calculation for the summed peptides belonging to the same protein. The ratios between the control and treated samples for each protein were obtained, a second normalization step for all the ratios to the median value was carried out. Log transformation of the normalized ratios and t-test (two-tailed) for significant differences between the control and treatment samples were conducted. The significant peptides labeled with iodoTMT were compared with the significant proteins quantified via iTRAQ. Student’s t-test was conducted between the fold change of the iodoTMT-labeled peptides and the fold change of the corresponding proteins quantified via iTRAQ. A correction factor was applied to the fold change of iodoTMT-labeled peptides, taking into account the fold change of the protein quantified via iTRAQ. The proteomics data were submitted to MASSIVE (MSV000084409) and can be accessed via username s_lawrence and password 797960sS!.

### 3.9. Functional Annotation and Hierarchical Clustering

We used Araport (https://apps.araport.org/thalemine) annotations, Panther GO (http://pantherdb.org/) tools, and Fisher’s Exact with false-discovery-rate to perform functional analysis of significant differentially redox-regulated proteins. For hierarchical clustering analysis the log (base2) transformed data, treatment and control ratios were used (http://bonsai.hgc.jp/∼mdehoon/software/cluster/software.htm). Using a tree algorithm, proteins were organized based on similarities in their expression profile. Short branches join proteins that are very similar to each other while longer branches join proteins that are less similar to each other.

## 4. Conclusion

We present the utility of iodoTMTRAQ in the identification and quantification of cysteine SNO modifications in an important plant single-cell type, guard cell. We identified 35 potential SNO-regulated guard cell proteins in response to the bacterial peptide elicitor flg22 after taking into consideration protein level changes. The nitrosylated proteins, most of which have not been identified in previous redox proteomic experiments, were largely involved in metabolism, stress and defense and may provide further insight to the importance of this oxidative PTM in guard cell immunity against pathogens. This study provides the first inventory of potential SNO-regulated proteins in stomatal guard cells. The action of NO in stomatal immunity against bacterial pathogens is not fully understood. For example, many protein targets of NO signaling in guard cells are still unknown. The identification of these targets will lead to a deeper understanding of how guard cells perceive and respond to NO, thus the results of this work are useful. Future studies using biochemical and genetic tools are necessary to functionally characterize the nitrosylated proteins revealed in this study. The result of future studies will provide insight to the biological relevance of these findings and improve the overall knowledge of NO signaling in stomatal innate immunity against bacterial pathogens.

## Figures and Tables

**Table 1:** S-nitrosylated proteins identified in guard cells after flg22 treatment for 15 min, 30 min and 60 min. The tick under DiANNA indicates the predicted cysteine residue involved in disulfide bond formation.

Protein Accession	Peptide	Protein Description	Fold Change	*p*-value (FDR-adj.)	Biological Function	DiANNA
***15 min after flg22 treatment***						
AT2G10940.1	NPPPGYTCSI	Lipid-transfer protein (LTP) II	4.72	0.016	Lipid binding	√
AT2G10940.1	ATCPIDTLK	LTP II	4.67	0.025	Lipid binding	√
AT3G23810.1	FDNLYGCR	S-adenosyl-L-homocysteine hydrolase 2 (SAHH2)	3.29	0.024	Carbon metabolic process	√
AT3G44310.2	IGAAICWENR	Nitrilase (NIT) 1	3.18	0.003	Nitrogen compound metabolic process	√
AT3G08560.1	IVCENTLDAR	Vacuolar H^+^-ATPase (VHA) E2	2.94	0.001	Hydrogen ion transport	√
AT1G78830.1	CLGYFYK	Curculin-like lectin protein (CLP)	1.91	0.020	Response to cytokinin, Stress response	√
AT1G78850.1	GLLGWDETCK	Mannose binding lectin (MBL) 1	1.90	0.010	Mannose binding	√
AT1G78850.1	SPSLASCDPK	MBL 1	1.67	0.039	Mannose binding	√
AT5G54270.1	WAMLGAFGCITPEVLQK	Chlorophyll a-b binding protein (CBP) 3	1.48	0.032	Photosynthesis	−
***30 min after flg22 treatment***	
AT2G10940.1	NPPPGYTCSI	LTP II	2.22	0.005	Lipid binding	√
AT5G17220.1	LYGQVTAACPQR	Glutathione S-transferase (GST) phi 12	1.53	0.049	Nitrogen compound metabolism, Stress response	√
AT1G26850.3	CLIPWGANDGMYLMEVDR	S-adenosyl-L-methionine- methyltransferase (SAMMT)	0.64	0.035	Methylation	√
AT5G54270.1	WAMLGAFGCITPEVLQK	CBP 3	0.57	0.047	Photosynthesis	−
AT1G78850.1	GLLGWDETCK	MBL1	0.55	0.023	Mannose binding	√
AT1G65590.1	VVPFEPGSCLAQ	Beta-hexosaminidase (HAD) 3	0.54	0.021	Lipid metabolic process	√
AT3G01500.1	VCPSHVLDFQPGDAFVVR	Beta-carbonic anhydrase (CA) 1	0.49	0.047	Carbon utilization, Stress response	√
AT4G39710.1	SGLGFCDLDVGFGDEAPR	FK506-binding protein (FKBP) 16-2	0.43	0.012	Photosynthesis	√
AT5G63800.1	SPDAPDPVINTCNGMK	Beta-galactosidase (GALD) 6	0.38	0.035	Carbohydrate metabolism	√
AT3G52500.1	YLCSGCDFSGLDPTLIPR	Aspartyl protease (APP)	0.30	0.039	Cellular process	√
AT4G28520.3	VVPGCAETFMDSQPMQGQQQGQPWQGR	Cruciferin (CRU) 3	0.15	0.045	Response to abscisic acid	√
AT5G44120.3	VIPGCAETFQDSSEFQPR	CRU 1	0.10	0.027	Response to abscisic acid	√
***60 min after flg22 treatment*** AT3G44310.2 AT3G44310.3	CIWGQGDGSTIPVYDTPIGK	NIT 1	6.31	0.049	Nitrogen compound metabolic process	√
AT3G23810.1	FDNLYGCR	SAHH2	3.29	0.024	Cysteine and methionine metabolism	√
AT4G11150.1	IVCENTLDAR	VHA E1	2.55	0.003	ATP synthesis	√
AT2G05520.1	QGGGGSGGSYCR	Glycine-rich protein (GRP) 3	2.12	0.028	Stress response	√
ATCG01060.1	CESACPTDFLSVR	PSI PsaC subunit	2.11	0.031	Photosynthesis	√
AT5G38410.3	QVQCISFIAYKPPSFTEA	Ribulose bisphosphate carboxylase (Rubisco) small chain (RBCS)	2.03	0.049	Photosynthesis	√
AT2G30970.1	IAAVQTLSGTGACR	Aspartate aminotransferase (APAT)1	1.99	0.023	Amino acid metabolic process	√
AT2G21060.1	ECSQGGGGYSGGGGGGR	GRP 2	1.96	0.030	Stress response	√
AT3G62940.3	LKPLGLTVSEIKPDGHCLYR	Cysteine proteinase (CP)	1.91	0.008	-	√
AT5G16390.1	QLDCELVIR	Acetylcoenzyme A carboxylase (AAC) 1	1.71	0.001	Fatty acid biosynthetic process	√
ATCG00490.1	VALEACVQAR	Rubisco Large chain (RBCL)	1.71	0.019	Photosynthesis	√
AT2G20360.1	YIQVSCLGASVSSPSR	NAD (P)-binding protein (NBP)	1.45	0.018	Electron transport, Stress response	−
AT5G08680.1	CALVYGQMNEPPGAR	ATP synthase subunit (ASS) beta-3	1.45	0.046	ATP synthesis	−
AT5G23890.1	VIETDTQPSDLCTR	Unknown protein (UNK)	1.45	0.007	−	√
AT3G09820.1	AGCYASNVVIQR	Adenosine kinase (ADK) 1	1.42	0.010	Purine metabolism, response to stimuli	√
AT5G03340.1	YTQGFSGADITEICQR	Cell division cycle (CDC) 48	1.30	0.041	Protein transport, cell division	√
AT4G13010.1	LANAHVTATCGAR	Oxidoreductase (OR)	1.28	0.046	Oxidation and reduction	√
AT1G42970.1	TNPADEECKVYD	Glyceraldehyde-3-phosphate dehydrogenase (GAPDH) B	0.79	0.031	Glucose metabolism, Oxidation and reduction	√
AT5G36700.1	ENPGCLFIATNR	2-phosphoglycolate phosphatase (PGP) 1	0.76	0.043	Photorespiration	√
AT3G01500.3	YMVFACSDSR	CA 1	0.69	0.021	Carbon utilization	−
AT5G14740.1	VLAESESSAFEDQCGR	CA 2	0.69	0.007	Carbon utilization	−

## Supplementary Materials

Supplementary Materials can be found at https://www.mdpi.com/1422-0067/21/5/1688/s1. Supplemental Table S1. List of proteins significantly changed in guard cells at 15 min after exposure to flg22. Supplemental Table S2. List of proteins significantly changed in guard cells at 30 min after exposure to flg22. Supplemental Table S3. List of proteins significantly changed in guard cells at 60 min after exposure to flg22.
