# Supplementary material for: S-Nitroso-Proteome Revealed in Stomatal Guard Cell Response to Flg22
Source: Int J Mol Sci. 2020 Mar 1;21(5):1688. doi: 10.3390/ijms21051688 (PMC7084773; doi:10.3390/ijms21051688)
Supplement: Supplementary file 1 [file ijms-21-01688-s001.pdf]

Supplemental Table 1. List of proteins significantly changed in levels in guard cells 15 min after exposure to flg22.

Protein Accession	Protein Description	Fold change	p-value
AT5G26000	Myrosinase 1	1.339	0.050
AT5G07440	Glutamate dehydrogenase 2	1.283	0.042
AT3G13930	Dihydrolipoyllysine-residue acetyltransferase component 2 of pyruvate dehydrogenase complex	1.280	0.040
AT4G35260	Isocitrate dehydrogenase [NAD] regulatory subunit 1	1.251	0.038
AT3G14310	Pectinesterase/pectinesterase inhibitor 3	1.243	0.038
AT1G29660	GDSL esterase/lipase	1.219	0.037
AT1G53240	Malate dehydrogenase 1	1.205	0.031
AT5G54270	Chlorophyll a-b binding protein 3	0.742	0.004
AT1G15820	Light harvesting complex photosystem II subunit 6	0.724	0.002

Supplemental Table 2. List of proteins significantly changed in levels in guard cells 30 min after exposure to flg22.

Protein Accession	Protein Description	Fold change	p-value
AT2G47000	ABC transporter B family member 4	1.913	0.007
AT2G44350	Citrate synthase 4	1.639	0.039
AT5G16390	Biotin carboxyl carrier protein of acetyl-CoA carboxylase 1	1.518	0.007
AT1G63000	Bifunctional dTDP-4-dehydrorhamnose 3	1.518	0.0173
AT1G65930	Cytosolic isocitrate dehydrogenase	1.467	0.039
AT5G20890	T-complex protein 1 subunit beta	1.457	0.002
AT3G09200	60S acidic ribosomal protein P0-2	1.430	0.001
AT5G14780	Formate dehydrogenase	1.411	0.019
AT1G72370	40S ribosomal protein Sa-1	1.386	0.028
AT1G13440	Glyceraldehyde-3-phosphate dehydrogenase GAPC2	1.385	0.016
AT3G25140	Galacturonosyltransferase 8	1.374	0.038
AT1G53240	Malate dehydrogenase 1	1.358	0.024
AT5G15490	UDP-glucose 6-dehydrogenase 3	1.351	0.006
AT1G01090	Pyruvate dehydrogenase E1 component subunit alpha-3	1.299	0.005

Supplemental Table 2. Continued

Protein Accession	Protein Description	Fold change	p-value
AT5G47200	Ras-related protein RABD2b	1.297	0.004
AT2G33150	3-ketoacyl-CoA thiolase 2	1.284	0.025
AT5G08590	Serine/threonine-protein kinase SRK2G	0.743	0.018
AT3G09810	Isocitrate dehydrogenase [NAD] catalytic subunit 6	0.732	0.003
AT4G26530	Fructose-bisphosphate aldolase 5	0.674	0.004
AT3G49120	Peroxidase 34	0.668	0.006
AT3G23400	Plastid-lipid-associated protein 6	0.654	0.016
AT1G15820	Light harvesting complex photosystem II subunit 6	0.642	0.011
AT3G15360	Thioredoxin M4	0.612	0.050
ATCG00720	Cytochrome b6	0.548	0.026
AT1G52400	Beta-D-glucopyranosyl abscisate beta-glucosidase	0.548	0.004
AT5G18170	Glutamate dehydrogenase 1	0.538	0.002
AT4G00370	Ascorbate transporter	0.534	0.014
ATCG00680	Photosystem II CP47 reaction center protein	0.514	0.001

Supplemental Table 3. List of proteins significantly changed in levels in guard cells 60 min after exposure to flg22.

Protein Accession	Protein Description	Fold change	p-value
AT4G03280	Cytochrome b6-f complex iron-sulfur subunit	1.436	0.033
AT5G26000	Myrosinase 1	1.422	0.005
AT5G52320	Cytochrome P450	1.414	0.018
AT4G25900	Galactose mutarotase-like superfamily protein	1.397	0.006
AT2G22480	ATP-dependent 6-phosphofructokinase 5	1.374	0.005
AT1G12900	Glyceraldehyde-3-phosphate dehydrogenase GAPA2	1.365	0.005
AT5G03660	Family of unknown function (DUF662)	1.347	0.022
AT1G53240	Malate dehydrogenase 1	1.331	0.011
AT2G45640	Histone deacetylase complex subunit SAP18	1.319	0.009
AT2G40610	Expansin-A8	1.312	0.008
AT1G20450	Dehydrin ERD10	1.290	0.002
AT2G20760	Clathrin light chain 1	1.278	0.005
AT5G49360	Beta-D-xylosidase 1	1.269	0.019
AT1G43160	Ethylene-responsive transcription factor RAP2-6	1.267	0.023

Supplemental Table 3. Continued

Protein Accession	Protein Description	Fold change	p-value
AT2G26040	Abscisic acid receptor PYL2	1.267	0.021
AT1G12270	Hsp70-Hsp90 organizing protein 1	1.263	0.005
AT5G15090	Mitochondrial outer membrane protein porin 3	1.260	0.010
AT4G32410	Cellulose synthase A catalytic subunit 1	1.257	0.013
AT3G45780	Phototropin-1	1.253	0.003
AT1G78830	EP1-like glycoprotein 2	1.245	0.029
AT5G48540	Cysteine-rich repeat secretory protein 55	1.242	0.041
AT5G16390	Biotin carboxyl carrier protein of acetyl-CoA carboxylase 1	1.241	0.009
AT2G41100	Calmodulin-like protein 12	1.240	0.004
AT1G07890	L-ascorbate peroxidase 1	1.231	0.048
AT4G39640	Gamma-glutamyltranspeptidase 1	1.226	0.001
AT4G22220	Iron-sulfur cluster assembly protein 1	1.216	0.002
AT2G33150	3-ketoacyl-CoA thiolase 2	1.214	0.003
AT3G20410	Calcium-dependent protein kinase 9	1.207	0.044

Supplemental Table 3. Continued

Protein Accession	Protein Description	Fold change	p-value
AT5G07440	Glutamate dehydrogenase 2	1.204	0.001
AT3G27380	Succinate dehydrogenase	1.203	0.001
AT5G49760	Leucine-rich repeat protein kinase family protein	1.202	0.030
AT2G21330	Fructose-bisphosphate aldolase 1	0.766	0.042
AT1G08450	Calreticulin-3	0.761	0.032
AT1G15820	Light harvesting complex photosystem II subunit 6	0.747	0.044
AT1G02870	Unknown function	0.734	0.030
AT2G16950	Isoform 2 of Transportin-1	0.718	0.001
AT2G29560	Cytosolic enolase 3	0.711	0.020
AT3G06350	Bifunctional 3-dehydroquinate dehydratase/shikimate dehydrogenase	0.707	0.014
AT1G19670	Chlorophyllase-1	0.678	0.035
AT1G52400	Beta-D-glucopyranosyl abscisate beta-glucosidase	0.490	0.030
AT3G57260	3-ketoacyl-CoA thiolase 2	0.373	0.028
